# Arquivos de Neuro-Psiquiatria: 80 years

**DOI:** 10.1055/s-0043-1764329

**Published:** 2023-03-14

**Authors:** Ayrton Massaro, Hélio A. G. Teive, Jose Antônio Livramento, Luis dos Ramos Machado, Paulo Caramelli

**Affiliations:** 1 Hospital Sirio-Libanês, São Paulo SP, Brazil.; 2 Universidade Federal do Paraná, Hospital de Clínicas, Departamento de Medicina Interna, Curitiba PR, Brazil.; 3 Universidade de São Paulo, São Paulo SP, Brazil.; 4 Universidade Federal de Minas Gerais, Faculdade de Medicina, Belo Horizonte MG, Brazil.

In June 2023, the Arquivos de Neuro-Psiquiatria (ANP) will complete 80 years of uninterrupted publication. The ANP was envisioned by the students of Professor Enjolras Vampré, founder of the São Paulo neurological school, who died prematurely. 1 The first edition of the ANP was in June 1943 and was presented by Professors Aderbal Tolosa (professor of Neurology at the University of São Paulo Medical School –USP), and Paulino Longo, (professor of Neurology at the Paulista School of Medicine – currently UNIFESP). 2 Dr. Oswaldo Lange (professor of Neurological Clinics at the University of São Paulo Medical School) was appointed editor-in-chief of the journal. Lange continued this noble work with mastery and dedication until 1986. 1 3 4 From 1987 to 2010, the editor-in-chief of ANP was Professor Antonio Spina-França, who was dedicated to making the ANP the leading journal of neurology in Latin America. 1 3 4 He was succeeded by his colleagues Professors Luís dos Ramos Machado and José Antonio Livramento, both from the Hospital das Clínicas at the University of São Paulo. They served as the editors-in-chief of the ANP between 2010 and 2017 with great harmony and dedication. 1 3 4 The editors chosen in 2018 were Professor Paulo Caramelli (full professor of Neurology at the University of Minas Gerais) and Hélio A. Ghizoni Teive (currently full professor of Neurology at the Federal University of Paraná). 1 3 4 This year, Professor Ayrton Massaro (Neurologist at the Hospital Israelita Albert Einstein, in São Paulo) replaced Professor Paulo Caramelli as editor-in-chief of the ANP. Demonstrating the great scientific evolution of Brazilian neurology in recent years, the ANP, the official journal of the Brazilian Academy of Neurology (BAN), has achieved an exponential increase in its impact factor, reaching 2.035. 5 This unprecedented achievement encourages even more its editors-in-chief, as well as all of its associated editors and the Brazilian neurological community, with the support of the BAN, to improve the scientific level of the journal, with the primary objective of reaching levels of excellence in the Latin American and world context of neurosciences. 5 This year, 2023, as current and past editors-in-chief of ANP, we are organizing a major scientific event to celebrate the 80th anniversary of the Journal, as well as the publication of a special issue of the ANP, with a series of scientific articles, covering topics of great interest in different subspecialties of neurology.
